# The value of using COVID-19 antibody tests as a potential approach to prioritize vaccination delivery

**DOI:** 10.1371/journal.pone.0311881

**Published:** 2024-10-16

**Authors:** Nasr Alrabadi, Haneen Obeidat, Razan Haddad, Noor Alyassin, Karem H. Alzoubi, Omar Obeidat, Saif M. Shteiwi, Daher Al-rabadi, Ibrahim Al-faouri

**Affiliations:** 1 Department of Pharmacology, Faculty of Medicine, Jordan University of Science and Technology, Irbid, Jordan; 2 Department of Medical Laboratory Sciences, Faculty of Applied Medical Sciences, Jordan University of Science and Technology, Irbid, Jordan; 3 Department of Pharmaceutical Sciences, Faculty of Pharmacy, Jadara University, Irbid, Jordan; 4 Department of Pharmacy Practice and Pharmacotherapeutics, University of Sharjah, Sharjah, United Arab Emirates; 5 Department of Clinical Pharmacy, Faculty of Pharmacy, Jordan University of Science and Technology, Irbid, Jordan; 6 Faculty of Medicine, The University of Jordan, Amman, Jordan; 7 Department of Community and Mental Health Nursing, Faculty of Nursing, Jordan University of Science and Technology, Irbid, Jordan; Waseda University: Waseda Daigaku, JAPAN

## Abstract

**Background:**

The highly contagious novel COVID-19 virus has demonstrated a great challenge for healthcare workers (HCWs) worldwide. One of these challenges is the availability of vaccines in some countries or societies, especially in the early stages of the pandemic.

**Objectives:**

This study aims to determine the level of natural immunity against COVID-19 infection among HCWs exposed to COVID-19 at the early stages of the pandemic and build a model to determine the groups that can benefit more from the scarce vaccination resources.

**Methods:**

This study took place between January and March 2021, after the first waves of the COVID-19 pandemic, before spreading the variants of concern, such as the UK variant (Alpha B.1.1.7), and before starting the vaccine campaigns. This cross-sectional study collected serum samples from 251 vulnerable HCWs. The samples were tested for IgG antibodies against COVID-19 using commercial kits. The demographics and clinical characteristics of the participants were recorded using face-to-face interviews.

**Results:**

COVID-19 IgG antibodies were detected in more than 40% of HCWs before vaccination. Those HCWs should have less priority than those without COVID-19 IgG. The seroprevalence of COVID-19 was higher in male HCWs and among nurses. There was no association between the participants’ immunity and smoking status or different blood groups. Most HCWs reported being infected with the virus during the first wave, mainly at the end of 2020. A limited number of HCWs reported infections between January 2021 and March 2021. All HCWs eventually received the COVID-19 vaccine, ignoring being previously infected.

**Conclusion:**

The reported results emphasize the value of using immunity tests to prioritize the groups that may benefit the most from the limited vaccines, especially in developing countries with scarce resources where those with COVID-19 IgG antibodies should have less priority for the COVID-19 vaccine. The present results indicate that up to 40% of the delivered vaccines to HCWs who had COVID-19 antibodies could be prioritized more wisely in future pandemics.

## Introduction

Coronavirus disease 2019 (COVID-19) is a highly contagious infectious disease caused by the SARS-CoV-2 virus, an enveloped, positive sense, single-stranded RNA coronavirus. It can spread directly via respiratory droplets, human-human transmission, and indirectly via contaminated objects [1–3]. Individuals infected with COVID-19 can be asymptomatic, present with mild-moderate respiratory symptoms such as fever, dry cough, and fatigue, or display more severe symptoms such as respiratory failure and shock [4–6].

By the end of 2019, COVID-19 became a worldwide threat and was labeled by the World Health Organization (WHO) as a pandemic in March 2020 [7, 8]. Epidemiologists worldwide worked diligently to find ways to study, monitor, and track the disease and develop guidelines to slow its spread and lessen its impact [9–13].

Some of the strategies that epidemiologists employed to control the spread of the pandemic included social distancing, country-wide quarantines, decreasing the duration of exposure to affected individuals, frequent hand washing with soap and water or hand sanitizer, the usage of personal protective equipment when in areas with possible exposure to the virus, and early detection of infection in affected individuals [1, 12–15].

In addition, scientists worldwide began developing vaccines to provide individual and population-wide immunity against the virus [16]. Due to the initial scarcity of vaccine availability, a framework was constructed for equitable allocation of the COVID-19 vaccine. Several factors were considered to form the framework, including the risk of acquiring COVID-19, the risk of severe morbidity and mortality, the risk of negative societal impact, and the risk of transmitting COVID-19 to others. As a result, some of the first recipients of the COVID-19 vaccine were high-risk healthcare workers (HCWs), first responders, and people with significant medical comorbidities. HCWs and first responders were included in the initial vaccination phase because of their crucial role in battling the pandemic and increased exposure to affected individuals [17–20]. Furthermore, seronegative individuals were prioritized over seropositive individuals due to the protective effects of the antibodies present in the serum of the seropositive individuals until their immunity waned [21].

This study aims to determine the prevalence of COVID-19 antibodies in HCWs and ascertain any significant associations between different variables among the HCWs and the presence of antibodies against COVID-19. Moreover, it aims to highlight the possibility of prioritizing the distribution of scarce vaccine resources to those who need them most or who can benefit more from them.

## Methodology

This cross-sectional study was performed at King Abdullah University Hospital in Jordan from January through March 2021. The hospital is an urban academic tertiary referral hospital located in the north of Jordan. It has 650 beds and serves five provinces in the region. The study protocol was approved by the hospital’s institutional review board (IRB) (IRB No. 130/136/2020).

The inclusion criteria were being a specialist HCW, including nurses, technicians, and administrative staff. There were no age, gender, or race restrictions. Excluded from the study were HCWs who had already been vaccinated before the study period. The study participants were chosen based on a convenience sampling approach. They were approached at their offices or clinics during lunch or rest breaks. Written consent was obtained from all the participants in the study. Questionnaires were handed out to the study participants. The survey included questions concerning the age, gender, occupation, ABO blood grouping, smoking status, period of infection with the COVID-19 virus, severity of symptoms during the time of infection, and past medical history of the participants. After the participants provided written informed consent, they filled out the questionnaires with the help of the investigators, and serum samples were drawn and tested for COVID-19 antibodies.

The participant’s serum was tested for antibodies against recombinant nucleocapsid protein using Elecsys Anti-SARS-CoV-2 S (Roche Diagnostics GmbH, Mannheim), a qualitative method of immunoglobulin detection. The overall specificity and sensitivity (≥14 days post-PCR) for the used kit were 99.95% (95% confidence interval [CI]: 99.87–99.99) and 97.92% (95% CI: 95.21–99.32) [22]. Cobas 6000 was used to incubate the samples in the biochemistry laboratory department [21, 23].

For the statistical analysis of the study’s data, version 25 of the Statistical Package for the Social Sciences (SPSS) software was utilized. The study’s data were summarized into frequencies and percentages. The chi-square test was used to detect associations among different data groups. P-values less than 0.05 were considered significant.

## Results and discussion

This study analyzed data from 251 HCWs. COVID-19 antibodies were detected in 117 out of 251 participants (46.6%) with a mean age of 35.7±10.3 years. Among the participants who reported previous COVID-19 infection (n = 107), 95 (81.2%) participants tested positive for COVID-19 antibodies, while 12 (9%) tested negative. Among the participants who reported symptoms while infected (n = 147), 89 (76.1%) tested positive for COVID-19 antibodies, while 58 (43.3%) participants tested negative. Of those who tested positive for COVID-19 antibodies and reported previous COVID-19 infection (n = 95), 52.6% (n = 50) reported the infection during November 2020, yet they were prioritized for COVID-19 vaccination.

A statistically significant association was found between antibodies in the participants’ serum and female gender (p = 0.017) and occupation (specialists p = 0.005, nurses p = 0.007). However, no statistically significant associations were found between COVID-19 antibodies and the participants’ age, area of residency, marital status, smoking status, and associated comorbidities (**Table 1**).

**Table 1:** Demographics of health care providers (HCWs), according to the presence of anti-COVID-19 antibodies in their serum samples.

	Absent (%)	Present (%)	Total (%)	P value
**N (%)**	134 (53.4)	117 (46.6)	251 (100)	0.812
**Male gender**	94 (70.1)	65 (55.6)	159 (63.3)	0.017
**Residents (Irbid)**	116 (86.6)	98 (83.8)	214 (85.3)	0.235
**Marital status**				
** Single**	44 (32.8)	34 (29.1)	78 (31.1)	0.521
** Married**	90 (67.2)	83 (70.9)	173 (68.9)	0.521
**Smoker**	38 (28.4)	22 (18.8)	60 (23.9)	0.077
**Occupation**				
** Specialist**	38 (28.4)	16 (13.7)	54 (21.5)	0.005
** Resident**	51 (38.1)	47 (40.2)	98 (39)	0.734
** Nurses**	24 (17.9)	38 (32.5)	62 (24.7)	0.007
** Technicians**	8 (6)	7 (6)	15 (6)	0.997
** Medical assistant**	12 (9)	6 (5.1)	18 (7.2)	0.243
** Administrator**	1 (0.7)	3 (2.6)	4 (1.6)	0.253
**Infected**	12 (9)	95 (81.2)	107 (42.6)	0
**Symptomatic**	58 (43.3)	89 (76.1)	147 (58.6)	0
**Co-morbidities**				
** Diabetic**	4 (3)	6 (5.1)	10 (4)	0.389
** Hypertension**	8 (6)	3 (2.6)	11 (4.4)	0.190
** Asthma**	1 (0.7)	4 (3.4)	5 (2)	0.132

The ABO blood group and Rh factor distribution in the healthcare workers who participated in the study stratified by the presence of COVID-19 antibodies are presented in **Table 2**. Among the participants who tested positive for COVID-19 antibodies, 40 were of blood group A, 22 were of blood group B, 40 were of blood group O, six were of blood group AB, 101 were Rh positive, and seven were Rh negative. No statistically significant association was found between the presence of COVID-19 antibodies and the ABO blood group and Rh factor distribution.

**Table 2:** The ABO blood group and Rh factor distribution in health care providers (HCWs), according to the presence of anti-COVID-19 antibodies in their serum samples.

	Absent (%)	Present (%)	Total (%)	P value
**A**	41 (30.6)	40 (34.2)	81 (32.3)	0.27
**B**	19 (14.2)	22 (18.8)	41 (16.3)	0.27
**O**	52 (38.8)	40 (34.2)	92 (36.7)	0.26
**AB**	6 (4.5)	6 (5.1)	12 (4.8)	0.26
**Rh**				
** Positive**	109 (81.3)	101 (86.3)	210 (83.7)	0.28
** Negative**	9 (6.7)	7 (6)	16 (6.4)	0.8

The first COVID-19 case reported in Jordan was on March 2^nd^, 2020 [24]. Since then and up to the time of conducting the study, the total number of cases recorded has almost reached a million cases, around 10% of the entire population of Jordan. This study analyzed the numbers and different factors concerning the presence or absence of COVID-19 antibodies in the serum of HCWs who work at one of the tertiary referral centers. This analysis indicated the numbers or the proportion of relatively immune or protective individuals. These factors include gender, category of healthcare worker, cigarette smoking and some comorbidities, previous COVID-19 infection and severity, ABO blood grouping, and Rh factor determination.

The present study included 159 males and 92 females. Among the male participants, 40.8% had positive antibody tests for COVID-19, while 56.5% of the female participants had positive results (OR of males to females (0.723, 95% CI; 0.558–0.937). Similar to this study, a study conducted in the USA reported a higher prevalence of COVID-19 in females (OR 2.6, 95% CI; 1.8–3.7) due to hormonal or genetic differences [25]. On the other hand, studies done in Sao Paolo, Brazil (38%, OR 1.56, 95% CI; 1.04–2.32), and Prishtina, Kosovo (OR 2.08, 95% CI; 1.20, 3.61) demonstrated an increased risk of COVID-19 among males. In addition, Studies conducted in Boston, MA, Ahmedabad, India, Southern Switzerland, Dera Ghazi Khan, Pakistan, and 11 African countries reported the lack of a statistically significant difference in the risk of COVID-19 based on sex [26–32].

With regards to the job title of HCWs and their risk of COVID-19, this study reported that nurses were found to be at increased risk of COVID-19, and specialists were found to be at decreased risk of COVID-19; OR 2.205 95% CI (1.226–3.966) and 0.400 95% CI (0.209–0.765), respectively. This result is compatible with a study conducted in China in which 53.4% of the HCWs who acquired COVID-19 were nurses [33]. Another study from Finland found that most COVID-19 among HCWs occurred in nursing assistants (53%) and nurses (17%) [34]. In addition, a study in Pakistan reported that doctors (OR 1.463, CI’; 1.365–1.568) were at increased risk of COVID-19 [35]. Moreover, a study conducted in 11 African countries reported that HCW in the operating room (OR 3.2, CI; 1.3–8.0), in the emergency department (OR 3.2, CI; 1.1–9.4), and in obstetrics and gynecology department (OR 19.3, CI, 2.0–183.4) were at higher risk of COVID-19 [32]. A study conducted in Prishtina, Kosovo, reported that physicians were at higher risk of COVID-19 than nurses (OR 0.52, 95% CI; 0.29–0.93) in opposition to this study [33]. Studies conducted in Southern Switzerland and Boston, MA, USA reported no significant differences among the categories of healthcare workers and their risk of acquiring COVID-19 [28, 30]. While most other studies included all physicians in one category, regardless of the level of exposure, this study considered specialists to be a separate category because they have less contact with patients than their residents. Upon reanalysis of the data, it was found that 53.8% of all the healthcare workers who tested positive were physicians (OR 0.590, CI; 0.354–0.983). In agreement with that, as a tertiary and teaching hospital, the participating physicians in our cohort are expected to have less contact with the patients and even with other HCWs. Most of the hands-on duties are conducted by their fellows and residents.

Although many initially assumed that cigarette smokers would be at increased risk of COVID-19, studies conducted in Jordan, Italy, China, and the USA have shown a significantly decreased risk of COVID-19 in smokers. They proposed that the mechanism of the reduced risk is through the anti-inflammatory effects of nicotine and the increase in nitric oxide, halting viral replication [23, 36]. Cigarette smoking, in this study, did not have a significant association with seropositivity, in agreement with a survey conducted in Dera Ghazi Khan, Pakistan (20.83%) [31]. However, a recent systemic review and meta-analysis indicated that exposure to tobacco cigarettes or nicotine may influence initial host-pathogen interactions and disease severity, which are linked to cytokine storm and acute respiratory distress syndrome [37].

Regarding the ABO blood group and Rh factor, different studies have reported conflicting results concerning the association between them and the risk of COVID-19. This study reported no significant association, which agrees with studies done in Bahrain, France, and the USA [38–40]. However, a comprehensive literature review reported that the O blood group decreased the risk of acquiring COVID-19 due to the presence of anti-A antibodies. These antibodies directly inhibit the adhesion of Spike protein/Angiotensin-Converting Enzyme II with their expressing cell lines [41].

Many factors were considered when considering vaccine allocation, as mentioned previously. During the early stages of the pandemic, Mayo Clinic was forced to sub-prioritize its staff into seven vaccination waves according to the risk of acquiring COVID-19. However, they did not consider the comorbidities of their staff due to employee privacy rights to prevent discrimination [42]. Other allocation frameworks, such as the CDC’s Advisory Committee on Immunization Practice (ACIP) and the National Academies of Sciences, Engineering, and Medicine (NASEM), prioritize healthcare workers equally, but they prioritize comorbidities differently. Furthermore, NASEM prioritizes the homeless and incarcerated over non-frontline workers [43, 44]. Another study delves into the advantages and disadvantages of lottery-based vaccine allocation. Still, it ultimately concludes that HCWs and essential workers such as nursing home aids, garbage collectors, and supermarket workers should be prioritized over the general population when scarce vaccines are available [45].

This study has some limitations, including the cross-sectional design, which does not allow for the establishment of a cause-effect relationship, the potential recall bias in self-reported data, and the generalizability of the findings to other settings or populations. Future longitudinal studies that track antibody persistence and investigate other factors influencing antibody presence or studies in different healthcare settings are warranted.

## Conclusions

Antibodies have a crucial role in the defense against infections, including COVID-19. Investigating the seroprevalence of COVID-19 antibodies assisted in the study of the epidemiology of this disease. It aided in the monitoring of the pandemic and the development of effective vaccines against this disease. Importantly, with limited vaccine resources in such pandemics, the presence of the COVID-19 IgG antibodies may indicate relatively immune individuals and, therefore, can aid in prioritizing the distribution of the limited available vaccines. According to the current study, up to 40% of HCWs could not have been considered a priority in receiving the vaccine as they had COVID-19 IgG antibodies. Thus, the vaccines could have been given to others in need and can be postponed for those HCWs. During pandemics, more research should be done to investigate the routes of spread and risk factors for acquiring COVID-19 or similar infections to build an applicable and efficient model for the wise use of the available vaccines.

## Supporting information

S1 File (PDF)
