# Supplementary material for: The value of using COVID-19 antibody tests as a potential approach to prioritize vaccination delivery
Source: PLoS One. 2024 Oct 16;19(10):e0311881. doi: 10.1371/journal.pone.0311881 (PMC11482680; doi:10.1371/journal.pone.0311881)
Supplement: S1 File — (PDF) [file pone.0311881.s001.pdf]

Reactive.Abs.ROCHE	Blood.type	Co.morbidities	DM	HTN	Asthma	Smoker
1	3	0	0	0	0	0
1	7	0	0	0	0	0
1	1	0	0	0	0	0
1	3	0	0	0	0	0
1	1	0	0	0	0	1
1	3	1	0	1	0	1
1	6	0	0	0	0	1
1	1	0	0	0	0	0
1		0	0	0	0	0
1	2	0	0	0	0	0
1	2	0	0	0	0	0
1	1	0	0	0	0	0
1	1	0	0	0	0	1
1	3	0	0	0	0	1
1	1	0	0	0	0	0
1	2	0	0	0	0	0
1	2	1	0	1	0	1
1	3	1	1	1	0	1
1	1	0	0	0	0	0
0	4	0	0	0	0	0
0	2	0	0	0	0	1
0	3	0	0	0	0	0
0	3	0	0	0	0	1
0	1	0	0	0	0	1
0		0	0	0	0	1
0	2	0	0	0	0	0
0		0	0	0	0	0
0		0	0	0	0	0
0		0	0	0	0	0
0		0	0	0	0	0
0		0	0	0	0	0
0	3	1	1	1	0	0
0	2	0	0	0	0	0
0	1	0	0	0	0	0
0	2	0	0	0	0	1
0	3	0	0	0	0	0
0	6	0	0	0	0	0
0	2	0	0	0	0	1
0		0	0	0	0	1
0	4	0	0	0	0	0
0	3	0	0	0	0	0
0	1	0	0	0	0	0
0	2	0	0	0	0	0

0		0	0	0	0	0
0		1	0	0	1	0
0	3	0	0	0	0	0
0	1	0	0	0	0	0
0	3	0	0	0	0	0
0	3	0	0	0	0	0
0	1	0	0	0	0	0
0	3	0	0	0	0	0
0	3	0	0	0	0	0
0	1	1	1	1	0	0
0	2	0	0	0	0	1
0	2	0	0	0	0	1
0	1	0	0	0	0	0
0	1	0	0	0	0	0
0	3	0	0	0	0	0
0	2	0	0	0	0	0
0	7	1	0	1	0	0
0	1	0	0	0	0	0
0	1	0	0	0	0	0
0	3	0	0	0	0	0
0	2	0	0	0	0	0
0	7	0	0	0	0	1
0	1	1	1	1	0	1
0	3	0	0	0	0	1
0	1	0	0	0	0	0
0	7	1	0	1	0	0
0		0	0	0	0	0
0	1	0	0	0	0	0
0	3	0	0	0	0	1
0	7	0	0	0	0	0
0	6	0	0	0	0	0
0	1	0	0	0	0	1
0	3	0	0	0	0	0
0	1	0	0	0	0	0
0	3	0	0	0	0	1
0	7	1	0	0	0	0
0	3	0	0	0	0	1
0	3	0	0	0	0	1
0	3	0	0	0	0	0
0	1	0	0	0	0	0
0	6	0	0	0	0	1
0	1	0	0	0	0	1
0	1	0	0	0	0	1
0	1	0	0	0	0	0

0	3	0	0	0	0	1
0	2	1	0	1	0	0
0	3	0	0	0	0	0
0	1	0	0	0	0	0
0	1	0	0	0	0	0
0	3	0	0	0	0	1
0	1	0	0	0	0	0
0		0	0	0	0	0
0	1	0	0	0	0	0
0	1	0	0	0	0	1
0	3	0	0	0	0	1
0	4	0	0	0	0	0
0	3	0	0	0	0	0
0		0	0	0	0	1
0		0	0	0	0	1
0		1	0	1	0	1
0	3	0	0	0	0	0
0	2	0	0	0	0	0
0	3	0	0	0	0	1
0	1	0	0	0	0	1
0	1	0	0	0	0	1
0	1	0	0	0	0	0
0	3	0	0	0	0	0
0	6	0	0	0	0	0
0	3	1	0	1	0	1
0	1	0	0	0	0	0
0	3	0	0	0	0	0
0	5	0	0	0	0	1
0	2	0	0	0	0	0
0	2	0	0	0	0	0
0	3	0	0	0	0	0
0	1	0	0	0	0	0
0	1	0	0	0	0	0
0	3	0	0	0	0	0
0	3	0	0	0	0	1
0	3	0	0	0	0	1
0	3	0	0	0	0	0
0	3	0	0	0	0	0
0	1	0	0	0	0	0
0	7	0	0	0	0	0
0	1	0	0	0	0	0
0	3	0	0	0	0	1
0	3	0	0	0	0	0
0	1	0	0	0	0	0

0	3	0	0	0	0	0
0	3	0	0	0	0	0
0	1	0	0	0	0	0
0	3	0	0	0	0	0
0	2	0	0	0	0	0
0	2	0	0	0	0	0
0	1	0	0	0	0	0
0	1	0	0	0	0	0
1		0	0	0	0	0
1	2	0	0	0	0	0
0	3	0	0	0	0	1
0	2	0	0	0	0	0
0	3	0	0	0	0	0
0	2	0	0	0	0	0
0		0	0	0	0	0
0	3	0	0	0	0	1
0	1	0	0	0	0	1
0	3	0	0	0	0	0
0	3	0	0	0	0	0
0	3	0	0	0	0	0
0	3	0	0	0	0	0
0	3	0	0	0	0	0
0		0	0	0	0	0
1	3	0	0	0	0	0
1	3	0	0	0	0	1
1	1	0	0	0	0	0
1	1	0	0	0	0	0
1	3	0	0	0	0	0
1	3	0	0	0	0	1
1	7	1	1	0	0	0
1	7	0	0	0	0	0
1		0	0	0	0	0
1	3	1	0	0	0	1
1		0	0	0	0	1
1	3	1	1	0	0	1
1	2	0	0	0	0	0
1	5	0	0	0	0	0
1	1	0	0	0	0	0
1	1	0	0	0	0	0
1	3	1	0	0	0	0
1	3	0	0	0	0	0
1	2	0	0	0	0	1
1		0	0	0	0	0
1	3	1	1	0	0	0

1		0	0	0	0	0
1	1	0	0	0	0	0
1	3	0	0	0	0	0
1	1	0	0	0	0	0
1	3	0	0	0	0	1
1		0	0	0	0	0
1	1	0	0	0	0	0
1	1	1	0	0	1	1
1	1	0	0	0	0	0
1	4	0	0	0	1	0
1	3	0	0	0	0	0
1	3	0	0	0	0	0
1	3	0	0	0	0	0
1	1	0	0	0	0	0
1	2	0	0	0	0	0
1	1	0	0	0	0	0
1	3	0	0	0	0	0
1	7	0	0	0	0	0
1	1	0	0	0	0	1
1	3	0	0	0	0	0
1	2	0	0	0	0	0
1	1	0	0	0	0	1
1	1	0	0	0	0	0
1	3	0	0	0	0	1
1	3	0	0	0	0	0
1	1	0	0	0	0	1
1	1	0	0	0	0	0
1	1	0	0	0	0	0
1	1	0	0	0	0	0
1	2	0	0	0	0	0
1	2	0	0	0	0	0
1	3	0	0	0	0	0
1	1	0	0	0	0	0
1	3	0	0	0	0	1
1	3	0	0	0	0	0
1	4	0	0	0	0	0
1	4	0	0	0	0	0
1	1	0	0	0	0	0
1	3	0	0	0	0	0
1		0	0	0	0	0
1	2	0	0	0	0	0
1	3	0	0	0	0	0
1		0	0	0	0	0
1	3	0	0	0	0	0

1	3	0	0	0	0	0
1	7	0	0	0	0	0
1	3	1	0	0	1	0
1	1	0	0	0	0	0
1	1	0	0	0	0	0
1	2	0	0	0	0	1
1	1	0	0	0	0	0
0	4	1	1	0	0	0
1	3	0	0	0	0	0
1	2	0	0	0	0	0
1	2	0	0	0	0	0
1	1	0	0	0	0	0
1	3	0	0	0	0	0
1	2	0	0	0	0	0
1	4	0	0	0	0	0
1	3	0	0	0	0	0
1	3	0	0	0	0	0
1	2	0	0	0	0	0
1	1	0	0	0	0	0
1	1	0	0	0	0	0
1	1	0	0	0	0	0
1	2	0	0	0	0	0
1	2	0	0	0	0	0
1	3	0	0	0	0	0
1	3	0	0	0	0	0
1	1	1	0	0	1	0
1	7	1	1	0	0	0
1	6	1	1	0	0	0
1	3	0	0	0	0	0
1	2	0	0	0	0	0
1	2	0	0	0	0	0
1	1	0	0	0	0	1

flue.like.sym	INFECTED	Vaccinated
0	0	1
0	0	0
0	0	0
1	0	0
1	0	0
0	0	1
0	0	0
0	0	0
1	0	0
1	0	0
0	0	0
1	0	0
1	0	1
0	0	1
1	0	0
0	0	1
0	0	0
1	0	1
0	0	0
1	0	0
0	0	0
0	0	0
0	0	0
1	0	0
1	0	0
1	0	0
0	0	0
0	0	0
0	0	0
0	0	0
0	0	0
0	0	0
0	0	1
0	0	1
0	0	0
0	0	0
0	0	0
1	0	0
1	0	0
0	0	0
0	0	0
1	0	0
1	0	0

1	0	0
0	0	0
0	0	0
0	0	0
1	0	0
1	0	0
1	0	0
0	0	0
0	0	0
1	0	0
0	0	0
1	0	0
1	0	0
1	0	0
1	0	0
1	0	0
1	0	0
1	0	0
0	0	0
1	0	0
1	0	0
0	0	0
1	0	0
1	0	0
1	0	0
0	0	0
1	0	0
1	0	0
0	0	0
1	0	0
0	0	0
0	0	1
1	0	0
0	0	0
0	0	1
0	0	0
1	0	0
1	0	0
1	0	1
0	0	1
0	0	0
0	0	0
1	0	0
0	0	0

0	0	0
1	0	0
0	0	0
0	0	0
0	0	1
1	0	1
0	0	1
1	0	1
1	0	0
0	0	1
1	0	0
0	0	1
0	0	1
1	0	1
1	0	0
0	0	0
0	0	0
0	0	1
0	0	0
0	0	0
0	0	0
0	0	0
0	0	1
1	0	1
0	0	1
0	0	0
0	0	0
0	0	0
0	0	1
0	0	1
0	0	0
0	0	1
0	0	1
0	0	1
0	0	0
1	0	0
0	0	0
0	0	0
1	0	0
1	0	0
1	0	0
1	0	0
0	0	1
0	0	0

0	0	0
0	0	0
0	0	0
0	0	0
1	0	0
1	0	0
0	0	0
0	0	0
0	0	1
0	0	0
1	0	0
1	1	0
1	1	0
1	1	0
1	1	0
0	1	0
1	1	0
1	1	0
1	1	0
0	1	0
1	1	0
1	1	0
0	0	0
1	1	0
1	1	0
1	1	0
1	1	0
0	0	1
1	1	0
1	1	0
1	1	0
1	1	0
1	1	0
1	1	0
1	1	0
1	1	0
1	1	0
1	1	0
1	1	0
0	1	0
1	1	0
1	1	0
1	1	0
1	1	0

0	1	0
1	1	0
1	1	0
0	1	0
1	1	0
1	1	0
1	1	0
1	1	0
1	1	0
1	1	0
1	1	0
1	1	0
1	1	0
1	1	0
1	1	0
0	1	0
1	1	0
1	1	0
1	1	1
1	1	0
0	1	0
1	1	0
1	1	0
1	1	0
1	1	0
1	1	0
1	1	0
1	1	0
1	1	0
0	1	0
1	1	0
1	1	0
1	1	0
1	1	0
1	1	0
1	1	0
1	1	0
1	1	0
1	1	0
0	1	0
0	1	0
1	1	0
0	1	0
1	1	0

1	1	0
1	1	0
1	1	0
1	1	0
1	1	0
1	1	1
1	1	0
0	1	0
1	1	0
1	1	0
1	1	0
0	1	0
1	1	0
1	1	0
1	1	0
1	1	0
1	1	0
0	1	0
0	1	0
0	1	0
1	1	0
1	1	0
1	1	0
1	1	0
1	1	0
1	1	0
1	1	0
1	1	0
0	1	0
1	1	0
1	1	0
1	1	0
